# Losartan protects human stem cell-derived cardiomyocytes from angiotensin II-induced alcoholic cardiotoxicity

**DOI:** 10.1038/s41420-022-00945-2

**Published:** 2022-03-28

**Authors:** Yuanxiu Song, Hongxia Li, Shuhong Ma, Min Zhu, Wen-jing Lu, Feng Lan, Ming Cui

**Affiliations:** 1 Department of Cardiology, Peking University Third Hospital, Beijing, 100191 China; 2 Department of Pathology, Zhuzhou Hospital Affiliated to Xiangya School of Medicine, Central South University, Zhuzhou, China; 3 Shenzhen Key Laboratory of Cardiovascular Disease, Fuwai Hospital Chinese Academy of Medical Sciences, Chinese Academy of Medical Sciences and Peking Union Medical College, Shenzhen, 518057 China; 4 State Key Laboratory of Cardiovascular Disease, Fuwai Hospital, National Center for Cardiovascular Diseases, Chinese Academy of Medical Sciences and Peking Union Medical College, Beijing, 100037 China

**Keywords:** Cardiomyopathies, Embryonic stem cells

## Abstract

Alcoholic cardiomyopathy (ACM) is a myocardial injury caused by long-term heavy drinking. Existing evidence indicates that high levels of oxidative stress are the key to pathological cardiomyopathy caused by long-term exposure to high concentrations of alcohol, while angiotensin II (AngII) and its type 1 receptor (AT1R) play an important role in excessive drinking. Whether oxidative stress-induced damage in ACM is related to AngII and AT1R is unclear, and the effects of alcohol on the electrophysiology of myocardial cells have not been reported. Most existing studies have used animal models. This study established an in vitro model of ACM based on human induced pluripotent stem cell-derived cardiomyocytes (hiPSC-CMs). The transcriptional profiling of alcohol treatment was performed by RNA-seq analysis. The role of oxidative stress, the expression of nicotinamide adenine dinucleotide phosphate oxidase (NOX), and the role of AngII and AT1R in the overactivation of oxidative stress were studied using fluorescent labeling, Western blotting, and high-content quantitative analysis. Real-time cell analysis(RTCA) and microelectrode array (MEA) were used to continuously monitor myocardial beating, observe the effects of alcohol on myocardial electrophysiological activity, and clarify the protective effects of the AT1R blocker losartan on ACM. We found that AngII and AT1R contribute to the effects of alcohol on the myocardium through oxidative stress damage, the mechanism of which may be achieved by regulating NOX.

## Introduction

Alcoholic cardiomyopathy (ACM) is a myocardial injury caused by long-term alcohol abuse, clinically characterized by dilated left ventricle, reduced ventricular wall thickness, and myocardial contractile dysfunction [1]. ACM ultimately develops into reduced left ventricular ejection fraction or even heart failure. The incidence of ACM is related to the daily level and duration of alcohol consumption: an alcohol intake of 80 g/day for more than 5 years significantly increases the risk of ACM [2]. Epidemiological data have shown that ACM represents 3.8–47% of all cases of nonischemic cardiomyopathy [3, 4]. However, no effective therapy is currently available for ACM.

Increasing experimental evidence showed that apoptosis and oxidative stress play an important role in pathological cardiomyopathy caused by exposure to large amounts of alcohol [5]. Reactive oxygen species (ROS) and reactive nitrogen species (RNS) play an important role in the initiation and progression of cardiovascular disease [6]. Studies have shown that angiotensin II (AngII) and AngII type 1 receptor (AT1R) significantly contribute to excessive alcohol consumption in dogs [7]. This study provides direct evidence that alcohol induces the activation of nicotinamide adenine dinucleotide phosphate oxidase (NOX) through the AngII–AT1R pathway, increasing ROS and the apoptotic response in mice [8]. However, all those procedures have been performed almost exclusively in animal models; no experiments have been performed on human cell models to investigate the underlying mechanism of ACM.

Recently, the establishment of disease models and drug screening using human induced pluripotent stem cell-derived cardiomyocytes (hiPSC-CMs) has become a hot topic in cardiovascular research [9]. The hiPSC-CM technology is usually not directly available from the original heart tissue. In addition to avoiding damage to the heart tissue caused by sampling, it can truly simulate the real state of human cardiomyocytes. With stable electrophysiology and contraction characteristics, hiPSC-CMs provide new insights into the molecular mechanism of cardiovascular diseases by simulating many phenotypes of human cardiomyocytes [10, 11]. In this study, we established a model of acute ACM to investigate the effects of alcohol on the structure, function, and electrophysiological activities of cardiomyocytes and its underlying mechanism using hiPSC-CMs.

## Results

### Alcohol induces cell viability and electrophysiological abnormalities in hiPSC-CMs

**Fig. 1:**
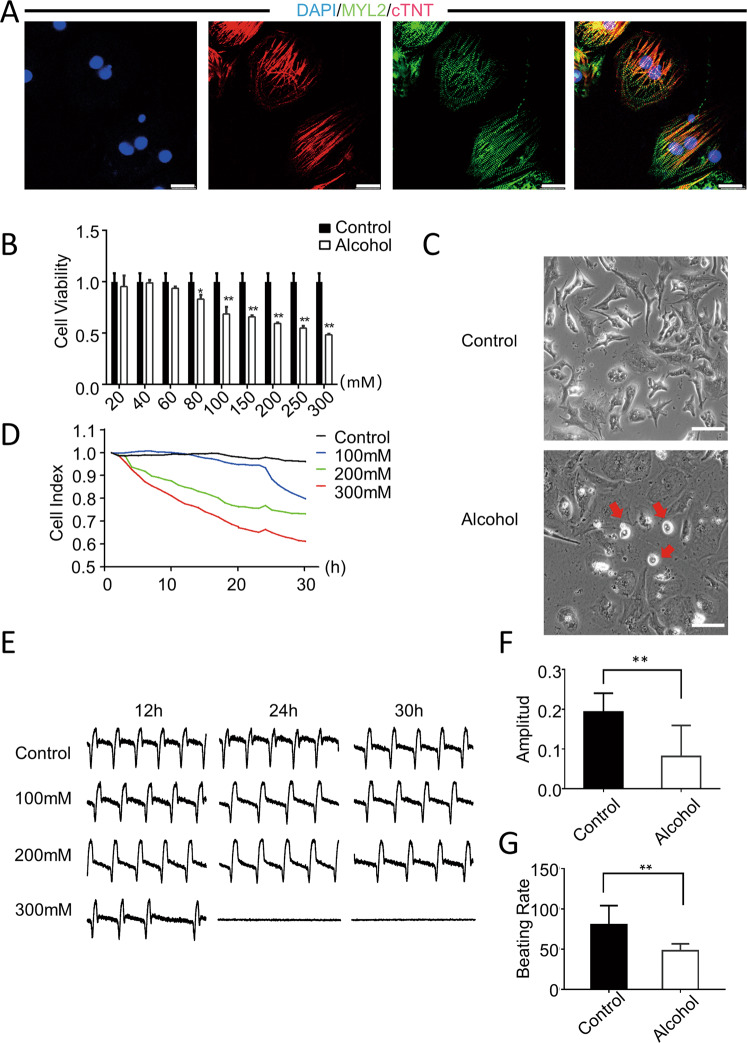
Modeling alcoholic cardiomyopathy using hiPSC-CMs. **A** Representative immunostaining for the expression of MYL2 (green) and cTNT (red) in hiPSC-CMs. Scale bar = 50 μm. **B** CCK-8 assay showed that the cell viability decreased to 95.3, 98.9, 93.6, 83.1, 68.5, 59.4, 54.8, and 48.3% after treatment with different concentrations of alcohol (i.e., 20, 40, 60, 80, 100, 150, 200, 250, and 300 mM) for 24 h (*n* = 4). **C** Representative images showed that hiPSC-CMs shrink and became round after alcohol treatment. Scale bar = 200 μm. **D** RTCA was used to monitor the changes in cell index after treating hiPSC-CMs with different concentrations of alcohol (i.e., 100, 200, and 300 mM) for 30 h (*n* = 30). **E**–**G** Changes in vibration amplitude and beat frequency over a 30-h period [**F**(*n* = 39); **G**(*n* = 41)]. Results are presented as means ± standard error of the mean (*P < 0.05, **P < 0.01, ***P < 0.001; two-sided Student’s *t*-test or one-way analysis of variance).

To establish a model of ACM, first, we used a small molecule-based protocol to differentiate hiPSC cell lines into hiPSC-CMs [12]. And the pluripotency of the hiPSCs was confirmed [13, 14]. Differentiated hiPSC-CMs stably expressed the myocardial atopic markers MYL2 and cTNT after induced differentiation (Fig. 1A). Subsequently, the hiPSC-CMs were used to establish a human cell model of ACM. The Cell-Counting Kit-8 (CCK-8) assay revealed that alcohol dose-dependently reduced cell viability, and cell viability showed a significant decline after exposure to more than 100-mM alcohol (Fig. 1B). The cardiomyocytes showed a shrunken and rounded morphology after exposure to hiPSC-CMs treated with 100-mM alcohol for 24 h (Fig. 1C).

To understand the electrophysiological changes in cardiomyocytes in alcoholic heart disease, we used real-time cellular analysis (RTCA) to dynamically monitor the effects of different concentrations (i.e., 0, 100, 200, and 300 mM) of alcohol on the electrophysiological activity and contractile function of hiPSC-CMs. The results showed that alcohol had a significant inhibitory effect on the contractility of hiPSC-CMs in a dose-dependent manner (Fig. 1D). Furthermore, alcohol has adverse effects on the beating rate and amplitude. The amplitude of hiPSC-CMs decreased after treatment with alcohol for 30 h (Fig. 1E, F). The beat rate of alcohol-treated hiPSC-CMs was also slower than that of controls (Fig. 1G).In addition, We examined the electrophysiological performance of cardiomyocytes using the microelectrode array (MEA). The results showed that under the treatment of 100, 200, and 400 mM alcohol, the field potential (FP) of cardiomyocytes was significantly prolonged and the contractility was significantly decreased. However, when alcohol was removed, the FP and contractility of cardiomyocytes with 100 and 200 mM alcohol could be recovered to a certain extent, while the FP and myocardial contractility of cardiomyocytes with 400 mM alcohol did not recover. This shows that the injury at a low concentration of alcohol is reversible, but the result of injury is irreversible after a high concentration of injury treatment. (Fig. S1A, B) These results demonstrated that alcohol affected the cell viability and electrophysiological activity of human cardiomyocytes.

### Alcohol induces cardiac cell apoptosis

**Fig. 2:**
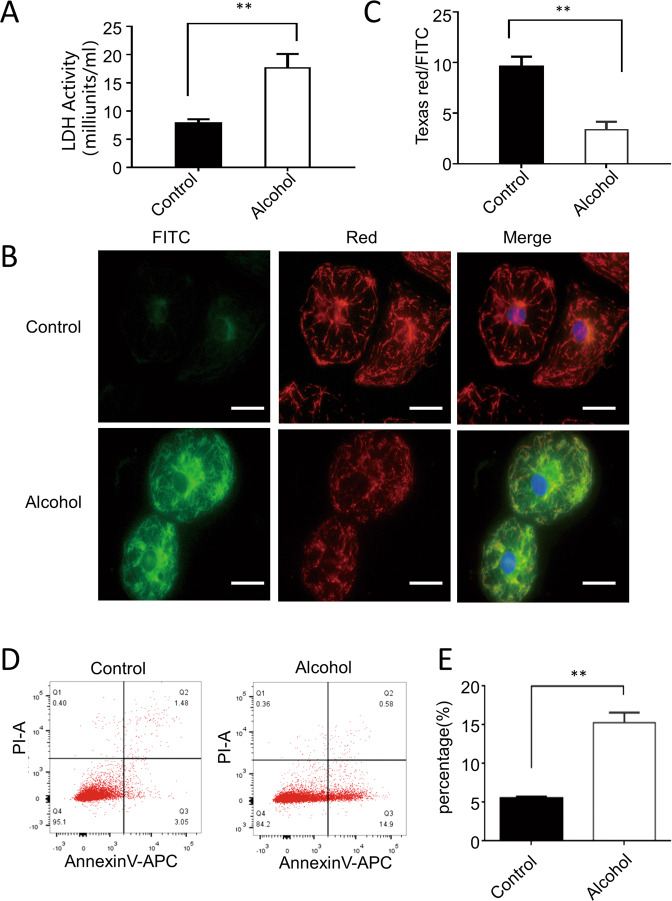
Alcohol-induced cardiomyocyte damage. **A** LDH activity assay showed that 100 mM alcohol can induce the upregulation of LDH expression in hiPSC-CMs 2.5 times (*n* = 7). **B** HTS analysis showed that the red-green fluorescence ratio has significantly dropped (*n* = 7). **C** The representative immunofluorescence image of JC-1 labeling showed that the green fluorescence expression of hiPSC-CMs significantly increased after treatment with 100-mM alcohol for 2 h. Scale bar = 25 μm. **D**, **E** Flow cytometry and quantitative analysis showed that 100-mM alcohol increased early apoptosis of hiPSC-CMs (*n* = 3). Results are presented as means ± standard error of the mean (**P < 0.01; two-sided Student’s *t*-test or one-way analysis of variance).

To further evaluate the cardiotoxicity of alcohol to hiPSC-CMs, we tested the release of lactate dehydrogenase (LDH) in hiPSC-CMs treated with 100-mM alcohol. Notably, alcohol caused obvious myocardial injury, as evidenced by an increment in LDH release (Fig. 2A). Moreover, mitochondrial membrane potential was evaluated using JC-1, which stains normally hyperpolarized mitochondria in red and damaged depolarized mitochondria in green. Confocal imaging showed that the red-to-green ratio has significantly dropped in hiPSC-CMs after alcohol treatment (Fig. 2B, C). This indicated that alcohol caused myocardial injury through mitochondrial damage, suggesting that cardiac apoptosis significantly contributes to alcohol-induced cardiotoxicity. Intriguingly, flow cytometry results showed a significant increase in apoptosis in hiPSC-CMs after exposure to alcohol (Fig. 2D, E).

### Analysis of transcriptional profile changes induced by alcohol in hiPSC-CMs

**Fig. 3:**
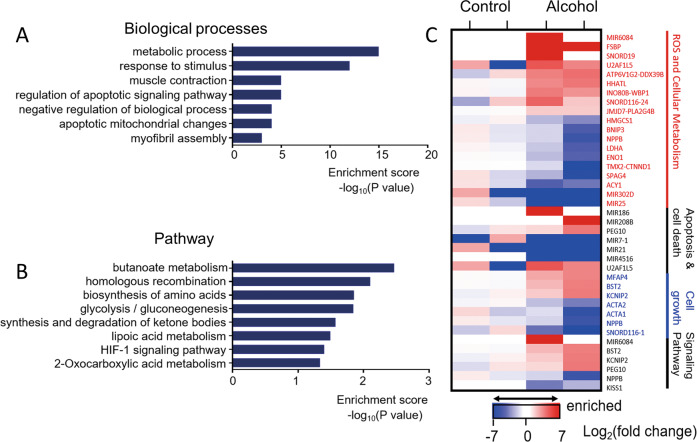
Alcohol-induced changes in the global gene expression profiles of cardiomyocytes. **A** The enriched terms GO (correlated *P* value of ≤0.05) of 100 mM alcohol treatment compared with vehicle. **B** The pathway analysis between the control and hiPSC-CMs treated with 100 mM alcohol is listed (*P* value of ≤0.05). **C** The heatmap of 39 differentially expressed RNAs between the 100 mM alcohol group and hiPSC-CMs showing four functional clusters. Each row corresponds to a single differentially expressed gene. Colors ranging from blue to red represent the relative expression levels (Log2(fold change)) of RNAs.

To explore the mechanism of myocardial injury induced by alcohol, RNA sequencing was performed on hiPSC-CMs treated with 100 mM alcohol. The results showed that 174 differentially expressed genes (DEGs) (more than onefold, probability of more than 0.8) were regulated by alcohol. Next, we performed gene ontology (GO) enrichment analysis of the 174 DEGs. These genes were enriched in seven biological processes, including metabolic process, response to stimulus, apoptotic signaling pathway, and apoptotic mitochondrial changes (Fig. 3A). Moreover, Kyoto Encyclopedia of Genes and Genomes (KEGG) analysis was performed to investigate the biological impacts of DEGs induced by alcohol. Eight pathways with significant DEGs showed significant differences between the alcohol-treated and control groups (Fig. 3B). These pathways were mainly related to butanoate metabolism, homologous recombination, biosynthesis of amino acids, and the HIF-1 signaling pathway, among others. Interestingly, among these DEGs, 19 were related to ROS and cellular metabolism, and seven were significantly related to cell death (Fig. 3C), which supported the aforementioned cell experimental results. These findings suggest that alcohol induces cardiac cell death by increasing metabolic-related ROS and apoptosis.

### Alcohol induces ROS and AngII/AT1R activation in cardiomyocytes

**Fig. 4:**
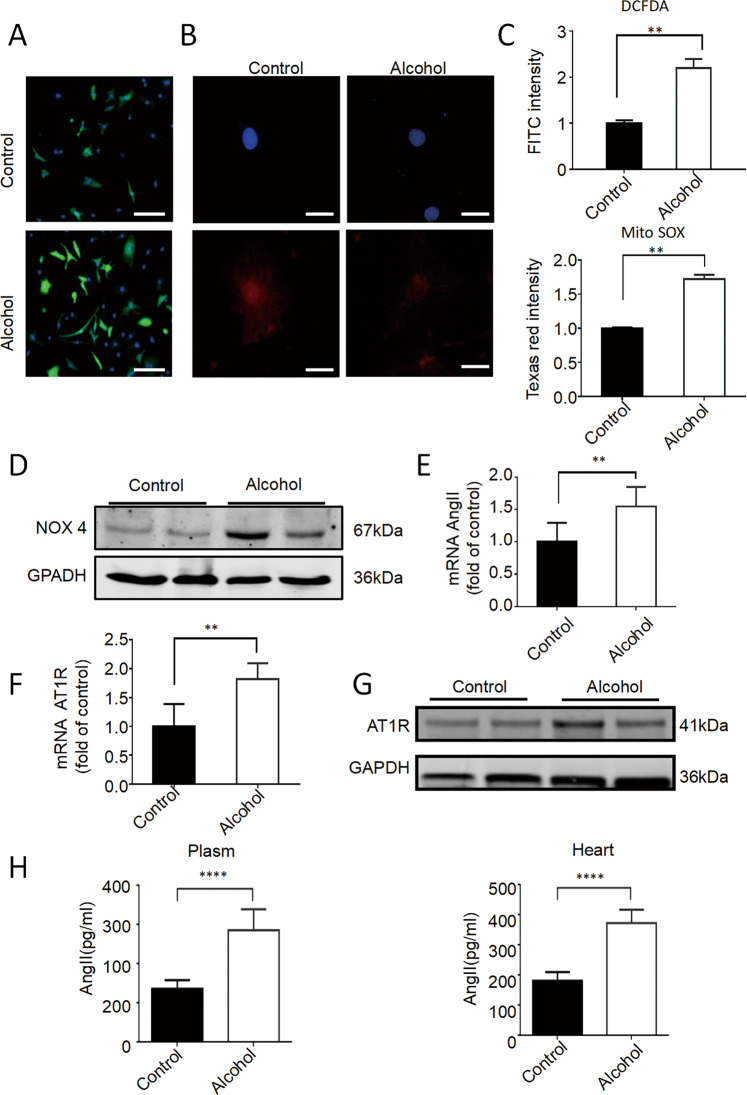
Alcohol-induced ROS and AngII/AT1R activation in cardiomyocytes. **A**–**C** Representative immunofluorescence images of CM-H2DCFDA (scale bar = 15 μm) and Texas red labeling (scale bar = 25 μm), and quantitative statistics showed that the level of the oxidative stress and mitochondrial oxidative stress significantly increased after hiPSC-CMs were treated with 100 mM alcohol for 2 h (*n* = 10). **D** Western blot showed that AT1R protein expression increased significantly after treatment with 100 mM ethanol for 24 h (*n* = 5). **E** RT-PCR showed that the expression of AngII and AT1R expression increased significantly after treatment with 100 mM alcohol for 24 h (*n* = 5). **F** RT-PCR showed that the expression of AngII and AT1R expression increased significantly after treatment with 100 mM alcohol for 24 h (*n* = 5). **G** Western blot showed that AT1R protein expression increased significantly after treatment with 100 mM alcohol for 24 h (*n* = 5). **H** Enzyme-linked immunosorbent assay showed that the level of AngII in the plasma and heart of mice increased after alcohol treatment (*n* = 6). Results are presented as means ± SEM (**P < 0.01; two-sided Student’s *t*-test or one-way analysis of variance).

To understand whether ROS plays an important role in alcohol-induced cardiac injury, we further investigated the effects of alcohol on ROS production. Confocal imaging and high-throughput screening (HTS) analysis showed that the fluorescence intensity in hiPSC-CMs treated with alcohol was significantly enhanced after staining with CM-H_2_DCFDA (index of oxidative stress) and Texas red (mitochondrial superoxide index) (Fig. 4A–C), indicating an increased oxidative stress level in cardiomyocytes. Moreover, Western blotting results showed that the expression of NOX4, a ROX-related oxidase that regulates ROS production, significantly increased in hiPSC-CMs after exposure to alcohol (Fig. 4D and S2A). These results suggested that alcohol increases ROS production.

Studies have shown that the activation of the renin-angiotensin system (RAS) was important for oxidative stress in many cardiovascular diseases [7]. Therefore, we investigated the role of AngII and AT1R (the key effectors for the RAS) in the hiPSC-CM model of acute ACM. Interestingly, real-time PCR analysis showed that the expression of AngII significantly increased in hiPSC-CMs after 100-mM alcohol treatment for 24 h (Fig. 4E), accompanied by a significant increase in the expression of AT1 both in the RNA and protein levels (Fig. 4F, G and S2B). Meanwhile, the secretion of AngII in the supernatant of hiPSC-CMs treated with alcohol significantly dose-dependently increased (Fig. S2C). In addition, AngII activation of AT1R activates promotes ROS production, resulting in increased phosphorylation of P38. The further results showed that the phosphorylation of P38 increased significantly in cardiomyocytes after alcohol treatment (Fig. S2D). Moreover, we determined the level of AngII in the plasma and heart tissue of mouse models of acute ACM. The results showed that the levels of AngII in the plasma and heart tissue significantly increased in alcohol-treated mice (Fig. 4H). These results suggest that alcohol activates the expression of AngII and AT1R, which can cause myocardial cell injury by activating the renin-angiotensin axis.

### Blocking AT1R prevents electrophysiological abnormalities and cardiac oxidative stress in the hiPSC-CMs model of ACM

**Fig. 5:**
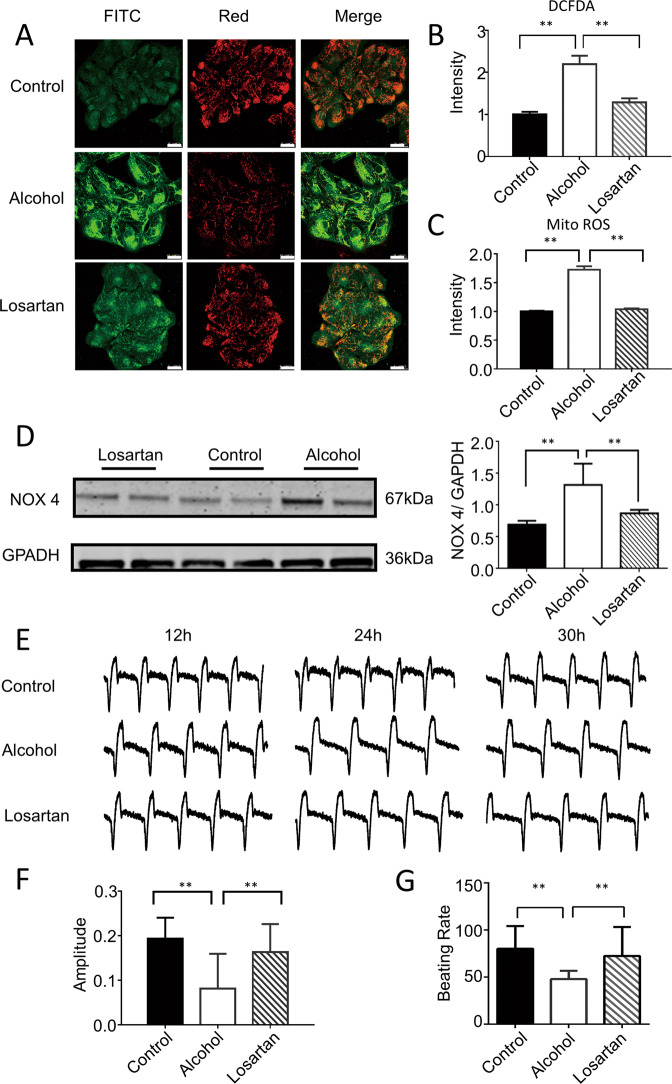
Blocking AT1R prevents the electrophysiological abnormalities and cardiac oxidative stress in hiPSC-CMs models of ACM. **A** Representative immunofluorescence images of JC-1 labeling showed a reduction in green fluorescence intensity after losartan administration compared with that after alcohol treatment (*n* = 20). Scale bar = 25 μm. **B** Representative immunofluorescence images of CM-H2DCFDA showed that losartan restored the level of mitochondrial oxidative stress induced by 100-mM alcohol. (*n* = 10) **C** Representative immunofluorescence images of Texas red labeling showed that losartan restored the level of the oxidative stress induced by 100-mM alcohol. (*n* = 10) **D** Western blotting and quantitative statistics showed that losartan restored the protein expression level of NOX4 induced by 100-mM alcohol (*n* = 5). **E**–**G** RTCA for dynamically monitoring cell index waveform, amplitude and beat frequency showed that losartan restored the electrophysiological disturbance of hiPSC-CMs induced by 100 mM alcohol [**F** (*n* = 39); **G** (*n* = 41)]. Results are presented as means ± standard error of the mean (**P < 0.01; two-sided Student’s *t*-test or one-way analysis of variance).

To confirm that AngII and AT1R are involved in the cardiotoxicity of alcohol, we used alcohol and losartan, an AT1R blocker, to co-treat hiPSC-CMs. RNA sequencing showed that pretreatment of hiPSC-CMs with losartan completely prevented alcohol-induced apoptosis signaling pathway and negative regulation of biological processes (Fig. S3A, B). Notably, the alcohol-induced increase in serum LDH was reversed in losartan-treated hiPSC-CMs (Fig. S3C). Similar regression changes in cell viability were observed in the hiPSC-CMs pretreated with losartan (Fig. S3D). Confocal imaging showed that losartan reduced the alcohol-induced increase in ROS levels in cardiomyocytes (Fig. 5A–C). Meanwhile, Western blotting results showed that blocking AT1R restored the increase in the expression of NOX4 induced by alcohol (Fig. 5D). These results indicated that blocking AT1R protects the myocardium from alcohol-induced damages by preventing cardiac apoptosis and oxidative stress.

Moreover, we used RTCA to examine whether losartan affects the alcohol-induced electrophysiological abnormalities in myocardial cells. The results showed that the decreased amplitude and beating frequency of cardiomyocytes induced by alcohol were restored in losartan-treated cardiomyocytes (Fig. 5E–G). Our data indicated that alcohol-induced cardiac apoptosis and oxidative stress are AT1-dependent.

## Discussion

**Fig. 6:**
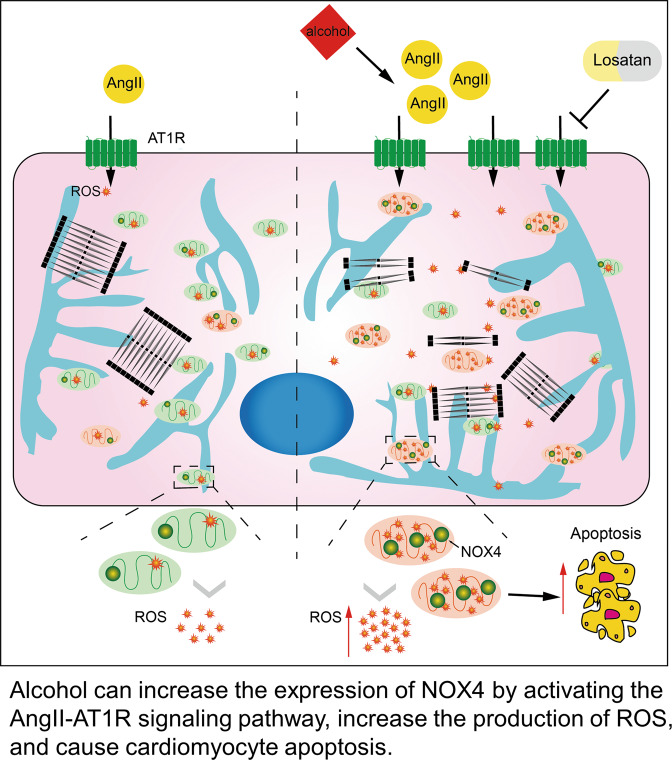
Schematic illustration of alcohol-induced cardiomyocyte damage in hiPSC-CMs. Alcohol can increase the expression of NOX4 by activating the AngII–AT1R signaling pathway, increasing the production of ROS, and causing cardiomyocyte apoptosis.

ACM is a heart disease caused by long-term excessive alcohol consumption. Even though the morbidity of ACM is increasing, there is a lack of criteria for the diagnosis of ACM, which is usually considered presumptive and is always excluded [15]. ACM models have been established almost exclusively using rodents; thus, the pathogenesis of human ACM is poorly understood. In this study, to investigate the underlying mechanism of human ACM, we used hiPSC-CMs to establish a model of acute ACM. Specifically, our findings demonstrated that AngII and AT1R are involved in the alcohol-induced cardiac damage by upregulating NOX4 and oxidative stress, leading to cardiomyocyte apoptosis (Fig. 6).

Currently, the pathogenesis of ACM is mainly explored using animal models. However, animal models have significant species differences from humans. Establishing human ACM cell models is essential for exploring the underlying pathogenesis of ACM. With the development of the hiPSC-CM technology, the human ACM model has become possible [9]. HiPSC-CMs is characterized by stable electrophysiological signals, regular beat rate, and normal cardiomyocyte function. More importantly, hiPSC-CMs overcome the species differences from other rodents [16]. An increasing number of cardiovascular disease models have been successfully established [17, 18]. In this study, we established a model of ACM based on hiPSC-CMs. We found that alcohol induces cell apoptosis, which is consistent with the findings of previous animal and clinical studies of ACM [19, 20]. Moreover, we found that alcohol reduces the CI, vibration amplitude, and beating frequency of hiPSC-CMs using the RTCA and MEA technology, which continuously and dynamically observes the effects of alcohol on the electrophysiological activity of hiPSC-CMs. This is the first study that has reported the effects of alcohol on myocardial electrophysiology.

ROS and RNS are involved in the occurrence and development of many cardiovascular diseases, such as hypertension, atherosclerosis, and ischemia-reperfusion injury [6, 21]. Although animal studies have shown that ACM was related to oxidative stress, ROS and RNS play an important role in the pathogenesis of human ACM [22, 23]. Consistent with the results of previous studies, our results showed that alcohol could increase cytoplasmic and mitochondrial oxidative stress levels in hiPSC-CMs. To further clarify the source of ROS, we also examined the expression of NOX, which can strictly regulate the production of ROS. Our results showed that alcohol induces oxidase stress by increasing the expression of NOX4, not NOX2, which are both abundantly expressed in the myocardium [24]. This is inconsistent with the findings of previous studies that have found that NOX2 is involved in regulating ACM [25]. The inconsistent results may be due to species differences and immaturity of hiPSC-CMs [26]. In future experiments, we also plan to use prolonged culture time, mechanical electrical stimulation, and other methods to promote the further maturation of hiPSC-CMs for further research [27]. Moreover, it is also necessary to knock down NOX4 in cardiomyocytes to verify that the effect of alcohol on cardiomyocytes depends on the expression of NOX4.

Many experimental studies have demonstrated that the RAS contributed to the occurrence and development of various diseases by regulating ROS [28]. In this study, we found that AngII and AT1R increased in hiPSC-CMs after exposure to alcohol, accompanied by an increase in NOX and ROS production, which is supported by the results of RNA sequencing, which showed that some DEGs were related to ROS. Moreover, losartan reduced the alcohol-induced increase in ROS and NOX4 levels in cardiomyocytes, preventing alcohol-induced cardiac apoptosis signaling pathways. These results suggested that AngII/AT1R was involved in alcohol-induced myocardial cell injury, leading to oxidase stress and cardiac apoptosis. This is consistent with previous findings that the increased myocardial NOX activity is closely related to the activation of RAS in hypertension and chronic heart failure [29–31]. These results suggest that blocking the RAS using losartan is a therapeutic intervention for alcohol-induced cardiotoxicity.

## Materials and methods

### Animal experiments

In this study, we used 12-week-old male C57BL/6 mice purchased from SPF (Beijing) Biotechnology Committee. The mice were placed in a 25 °C area with a 12:12-h light-and-dark cycle and fed with free rodent chow and tap water. After adaptive feeding for 7 days, the mice were randomly divided into the control and alcohol-treated groups, with six mice in each group. Before the experiment, the mice fasted and continuously drank water for 12 h. Ethanol was diluted to 50% in sterile water, and the mice in the alcohol-treated group were given the drug with a no. 18 intragastric tube (5 g/kg). The control group received the same volume of distilled water. The animals were euthanized 6 h after treatment, and peripheral blood and heart tissue were collected for subsequent experiments.

### The hiPSC culture

The hiPSC line was purchased from Cellapy (Beijing, China), which is an internationally recognized hESC (H9) cell line. The cells were tested by STR and mycoplasma kit, and no mycoplasma infection was found (Fig. S5A, B). Cells were routinely maintained in the presence of PSCeasy iPSC medium modified essential 8 medium (Cellapy, China) on six-well plates (Corning, USA) coated with a 1:200 dilution of Matrigel (Corning, USA). The medium was changed every other day and passaged every 3–4 days with 0.5 mM EDTA (Cellapy, China). The cells were grown in a humidified atmosphere of 95% air and 5% CO_2_ at 37 °C.

### Cardiac differentiation of hiPSCs

The hiPSCs were kept in a PSCeasy medium (Cellapy, China) and differentiated when they reached 70–80% confluence. The medium was changed to the basal differentiation medium, consisting of RPMI 1640 medium (Life Technologies, USA), B27 supplement (Life Technologies, USA), and 213-µg/mL l-ascorbic acid 2-phosphate (Sigma, USA). The medium was changed every other day (48 h). From day 0 to day 2, the medium was supplemented with 6-µM CHIR99021 (Selleck Chemicals, USA) and 25-ng/mL Activin A (Cellapy, China). On day 2, the medium was changed to the basal differentiational medium supplemented with 5 µM IWR-1 (Selleck Chemicals, USA). The medium was changed on day 4 and every other day for the basal differentiational medium. Contracting cells were noted from day 9.

### Drug treatment

For treating hiPSC-CMs, 100-mM alcohol was diluted in B27, and cells were treated for different durations. Then, 1 μM losartan (Selleck, USA) was diluted in B27, and hiPSC-CMs were co-treated with alcohol.

### Immunofluorescent staining (IF) and imaging analyses

The hiPSC-CMs were plated on 20-mm coverslips coated with 1:200 dilutions of Matrigel and were fixed with 4% paraformaldehyde(PFA) for 15 min. Then, after washing with phosphate-buffered saline (PBS) thrice for 10 min, the cells were permeabilized with 0.2% Triton X-100 (Sigma, USA) for 15 min and blocked with 3% bovine serum albumin(BSA) (Sigma, USA) for 1 h at room temperature. Then, the cells were incubated with primary antibodies, including OCT4 and SSEA 4 (Santa Cruz, USA), overnight at 4 °C. Moreover, cells were washed with PBS and incubated for 1 h at room temperature in the dark with secondary antibodies (Invitrogen). The cells were washed again as above, mounted with Fluoroshield Mounting Medium with 4,6-diamino-2-phenylindole. Images were taken under a confocal microscope (Leica DMI 4000B, German). The antibodies and their appropriate dilutions are provided in Table S2.

### Western blotting

Proteins from hiPSC-CMs were extracted using a Protein Extraction Kit (Promega, USA). The protein concentration of the supernatant was measured using the BCA method. Then, 20 µg protein was separated on 10% SDS-PAGE and transferred to PVDF membrane at 300 mA for 90 min, which was blocked with 5% albumin bovine at room temperature for 1 h and then incubated at 4 °C overnight with the primary antibodies GAPDH, AT1R, and NOX4 (Santa Cruz, USA) and then with IR dye-conjugated secondary antibodies (Rockland Immunochemical, USA) for 1 h at room temperature. GAPDH was used as an internal control. Blots were exposed and analyzed using an Odyssey infrared imaging system (LI-COR Biosciences, USA). The antibodies and their appropriate dilutions are shown in Table S2.

### Flow cytometry

The hiPSC-CMs under different treatments were singularized using CardioEasy CM dissociation buffer (Cellapy, China). The cells were incubated at 37 °C for 20–30 min. We observed that most clones were detached from the bottom of the plate under a microscope and gently pipetted the cells, sucked them out, centrifuged, and washed thrice with PBS. The cells were stained with Annexin V/PI kit (Invitrogen, USA), filtered through a 300-mesh filter, and immediately analyzed using FACS (Beckman, USA). The cell count is 2–3 million. The results were analyzed using the Flow Jo X program.

### RNA extraction and RT- PCR

Total RNA from hiPSC-CMs was extracted using TRIZOL Reagent (Invitrogen, USA). Then, 2 µg total RNA was reversed to cDNA using the GoScript Reverse Transcription System (Promega, USA). Quantitative RT-PCR involved the use of SYBR Green II (Takara, Japan) in the iQ5 system (Bio-Rad, Hercules, CA). The sequences of the primers were as follows: AngII, AT1R, NOX2, and GAPDH. A comparative computed tomography method was used to analyze the relative changes in gene expression. The results were expressed as relative to the data of GAPDH transcripts (internal control). Primer sequences are listed in Table S1.

### Enzyme immunoassay

The supernatant of hiPSC-CMs after different treatments was collected, and the concentration of AngII in the supernatant was measured using Human Angiotensin II ELISA Kit (Bio-Medical Assay, China). Peripheral blood plasma and tissue homogenate of mouse heart were collected from different treatment groups, and the concentration of AngII was measured using Human Angiotensin II ELISA Kit (Bio-Medical Assay, China).

### Measurement of cell viability

The cell viability was examined using Cell-Counting Kit-8 (CCK-8) assay in accordance with the manufacturer’s instructions. Briefly, 30,000–40,000 hiPSC-CMs were seeded in 96-well plates. Four days after beating, 90-µL medium and 10 µL CCK-8 (Dojindo, Japan) reagents were added to wells under different treatments and incubated for 2 h at 37 °C. The optical density (OD) of each well was measured at 450 nm using an automated microplate reader (BioTek, USA).

### Measurement of LDH activity assay

The LDH activity assay was used as a qualitative index of alcoholic cytotoxicity. The LDH concentration in the culture medium was examined using Lactate Dehydrogenase Activity Assay Kit (Sigma-Aldrich, USA). After different treatments of hiPSC-CMs, serum LDH activity was measured according to the manufacturer’s instructions.

### Measurement of ROS assay

To evaluate ROS production in hiPSC-CMs, oxidant-sensitive fluorescent probe/5-(and-6)-chloromethyl-2,7-dichlorodihydrofluorescein diacetate acetyl ester (CM-H_2_DCFDA) (Invitrogen, USA), a cell-permeant indicator, was used. After hiPSC-CMs were treated/incubated with CM-H_2_DCFDA at 37 °C for 30 min under different treatments, the dye was removed and washed once with PBS. Fluorescence was measured at 483-nm excitation and 520-nm emission using HTS and analyzed using MetaXpress High Content Image Acquisition.

### Measurement of JC-1

5,5′,6,6′-tetrachloro-1,1′,3,3′-tetraethylbenzimidazol-carbocyanine iodide (JC-1) staining Molecular Probes (Abcam, USA) were used as an indicator of early apoptosis, which can detect mitochondrial membrane potential, a fundamentally important determinant of cell function and health. Consequently, mitochondrial depolarization is indicated by a decrease in the red/green fluorescence intensity ratio. After different treatments, of hiPSC-CMs in 24-well culture plates were incubated with 5-µM JC-1 for 15 min at 37 °C and scanned using HTS; the red/green fluorescence intensity ratio was analyzed using MetaXpress High Content Image Acquisition (ACEA biosciences, USA).

### Statistical analysis

Results are expressed as mean ± standard deviation. All statistical analyses were performed using GraphPad Prism 6.00 for Windows. A two-sided unpaired Student’s *t*-test was used to compare two groups with normal distribution. One-way analysis of variance was used to compare three or more groups. All tests for normality and homogeneity of variance were passed before the *t*-test and one-way analysis of variance. *P* values of less than 0.05 were used to denote statistical significance.

## Supplementary information

Supplementary materials

## Data Availability

The RNA-seq data that support the findings of this study are available in the National Center for Biotechnology Information Gene Expression Omnibus (GEO) with the accession ID GSE195981 and can be found below: All software resources used in this study are indicated above and shown in the Key Resources Table.
