# Supplementary material for: Losartan protects human stem cell-derived cardiomyocytes from angiotensin II-induced alcoholic cardiotoxicity
Source: Cell Death Discov. 2022 Mar 28;8:134. doi: 10.1038/s41420-022-00945-2 (PMC8960777; doi:10.1038/s41420-022-00945-2)
Supplement: Supplementary file 1 — Supplementary materials [file 41420_2022_945_MOESM1_ESM.pdf]

Supplementary Figure legends

Supplementary Figure 1 Changes of electrophysiological activity of cardiomyocytes induced by alcohol at different concentrations

A.MEA showed changes in FP of cardiomyocytes treated with 100, 200 and 400mM alcohol.**B**.MEA showed changes in contractility of cardiomyocytes treated with 100, 200 and 400mM alcohol.

Supplementary Figure 2 Alcohol induced NOX4 upregulation and AngII/AT1R activation in cardiomyocytes. A.The expression of AT1R protein was determined by western blot after treatment with 100mM alcohol for 24 h. **B**.The expression of NOX4 protein was determined by western blot after treatment with 100mM alcohol for 24 h. **C**. Enzyme-linked immunosorbent assay (ELISA) showed that the level of AngII increased in hiPSC-CMs culture medium after alcohol treatment in a dose dependent manner. **D**.Western blot showed that phosphorylated P38 increased significantly after treatment with alcohol .

Supplementary Figure 3 losartan prevented alcohol-induced apoptosis in hiPSC-CMs. **A**.The heatmap showed genes that are significantly regulated with or without losartan after 100mM alcohol treatment of hiPSC-CMs. **B**.The enriched terms GO showed that losartan restored the increase in apoptosis signaling pathways and negative regulation of biological processes induced by 100 mM alcohol . **C**. LDH activity assay (below, n=7) showed that losartan restored the cell damage induced by 100 mM alcohol. **D**. The CCK8 assay (above, n=4) showed that losartan restored the decreased cell viability induced by 100 mM alcohol.

Supplementary Figure 4 Unclipped image of western blotting of each figure

Supplementary Figure 5 Identification of hiPSC cell lines. A. Mycoplasma testing revealed that the cell line was not infected with mycoplasma.**B.**STR detection in hiPSC cell lines.

Supplementary Tables

Supplementary Table 1: Primer sequences used for q-PCR

Gene	Forward primers（5′-3′）	Reward primers（5′-3′）
NOX2	TCACTTCCTCCACCAAAACC	GGGATTGGGCATTCCTTTAT
NOX4	CTTCCGTTGGTTTGCAGATT	TGGGTCCACAACAGAAAACA
AT1R	GAATATTTGGAAACAGCTTGGT	CAAAGTCAGTAAAAAGCATAAG
AngII	GCTAAGGACCCCACTGTTGCTA	TGTAGATGCCATTCGTGGTGTG
GAPDH	CAATGACCCCTTCATTGACC	GACAAGCTTCCCGTTCTCAG

Supplementary Table 2: Antibodies for immunofluorescence, flow cytometry and western blot in this study

Antibody	Application	Dilution	Manufacturer	Catalog
Anti-OCT4	IF	1:100	Santa Cruz	sc-9081
Anti-SSEA4	IF	1:100	Santa Cruz	sc-21706
Anti-cTnT	IF	1:100	Abcam	ab8295
	FC	1:200		
Anti-AT1R	WB	1:500	Santa Cruz	sc-515884
Anti-NOX4	WB	1:500	Santa Cruz	sc-518092
Anti-GAPDH	WB	1:500	Abcam	ab181602
Goat anti-Mouse IgG Alexa Fluor 594	IF	1:200	Invitrogen	A21145
Goat anti-Rabbit IgG Alexa Fluor 488	IF	1:200	Invitrogen	A32731
Goat anti-Rabbit IgG (H + L) IRDye 800CW	WB	1:20000	LI-COR	926-32211
Goat anti-Mouse IgG (H + L) IRDye 800CW	WB	1:20000	LI-COR	926-32211

IF: Immunofluorescence
FC: Flow cytometry
WB: Western Blot

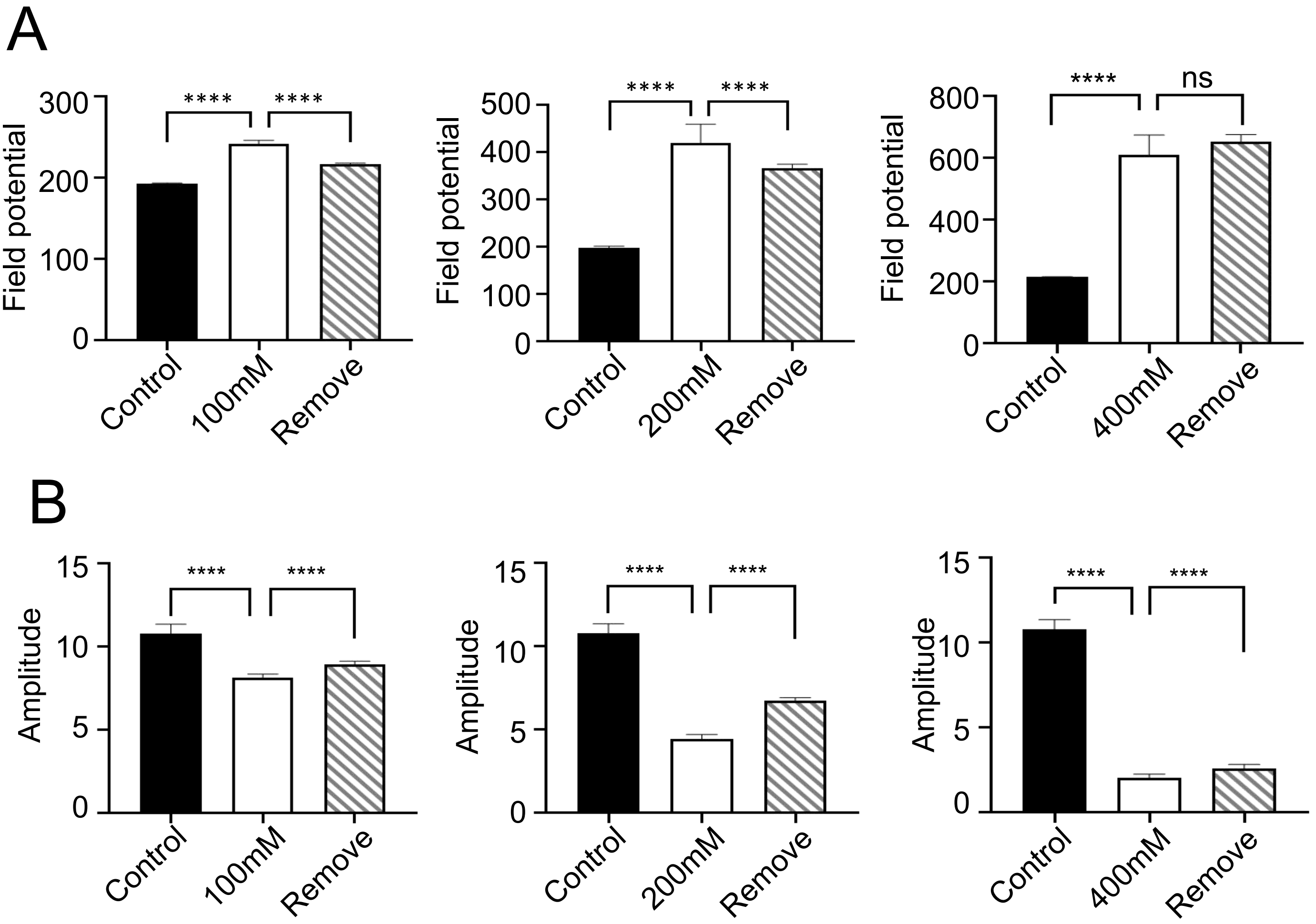
A
Field potential
300
200
100
0
****
****
Control
100mM
Remove
Field potential
500
400
300
200
100
0
****
****
Control
200mM
Remove
Field potential
800
600
400
200
0
****
ns
Control
400mM
Remove
B
Amplitude
15
10
5
0
****
****
Control
100mM
Remove
Amplitude
15
10
5
0
****
****
Control
200mM
Remove
Amplitude
15
10
5
0
****
****
Control
400mM
Remove

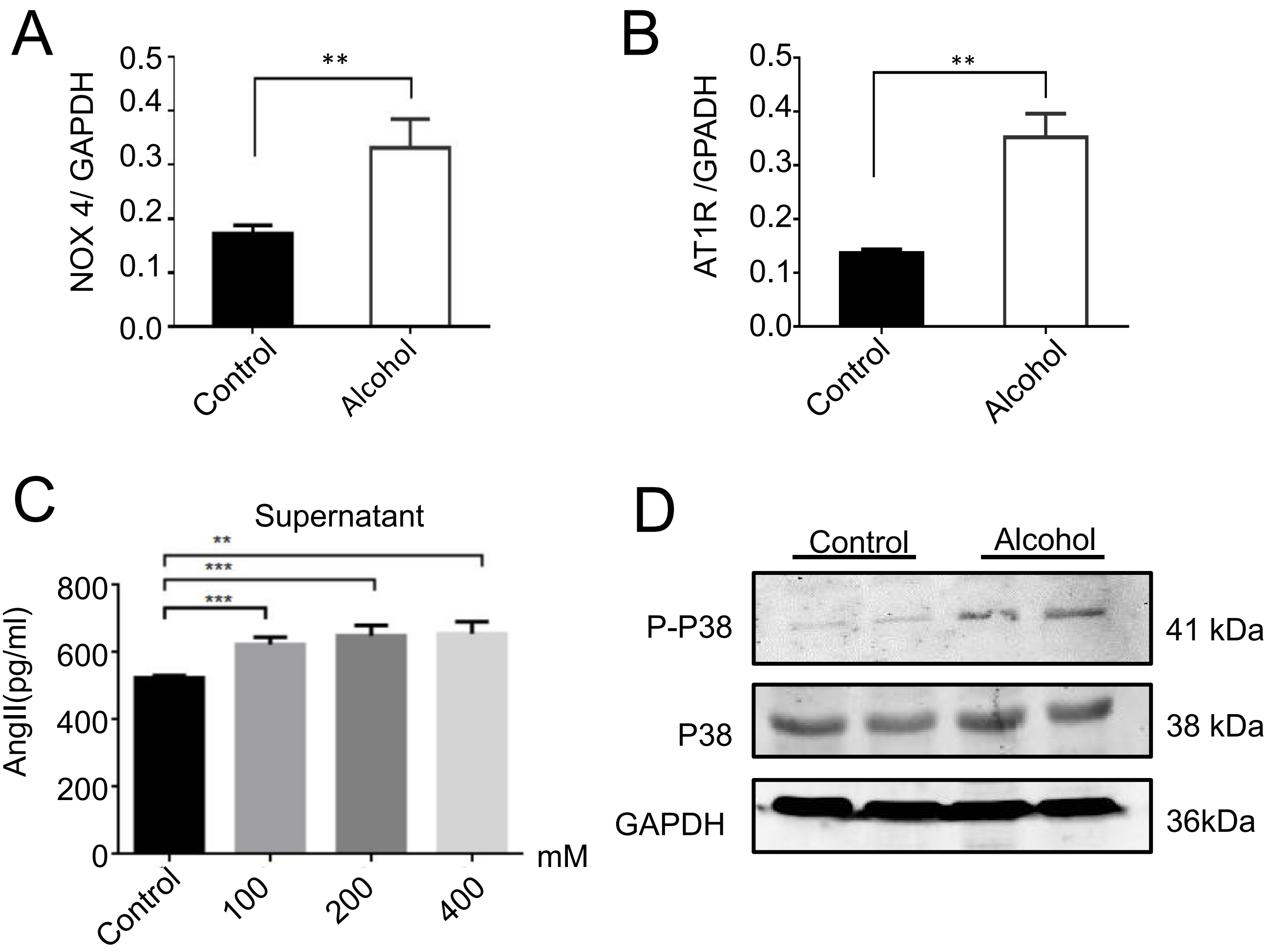
A
NOX 4/ GAPDH
0.5
0.4
0.3
0.2
0.1
0.0
**
Control
Alcohol
B
AT1R /GPADH
0.5
0.4
0.3
0.2
0.1
0.0
**
Control
Alcohol
C
Supernatant
AngII(pg/ml)
800
600
400
200
0
**
***
***
Control
100
200
400
mM
D
Control
Alcohol
P-P38
41 kDa
P38
38 kDa
GAPDH
36kDa

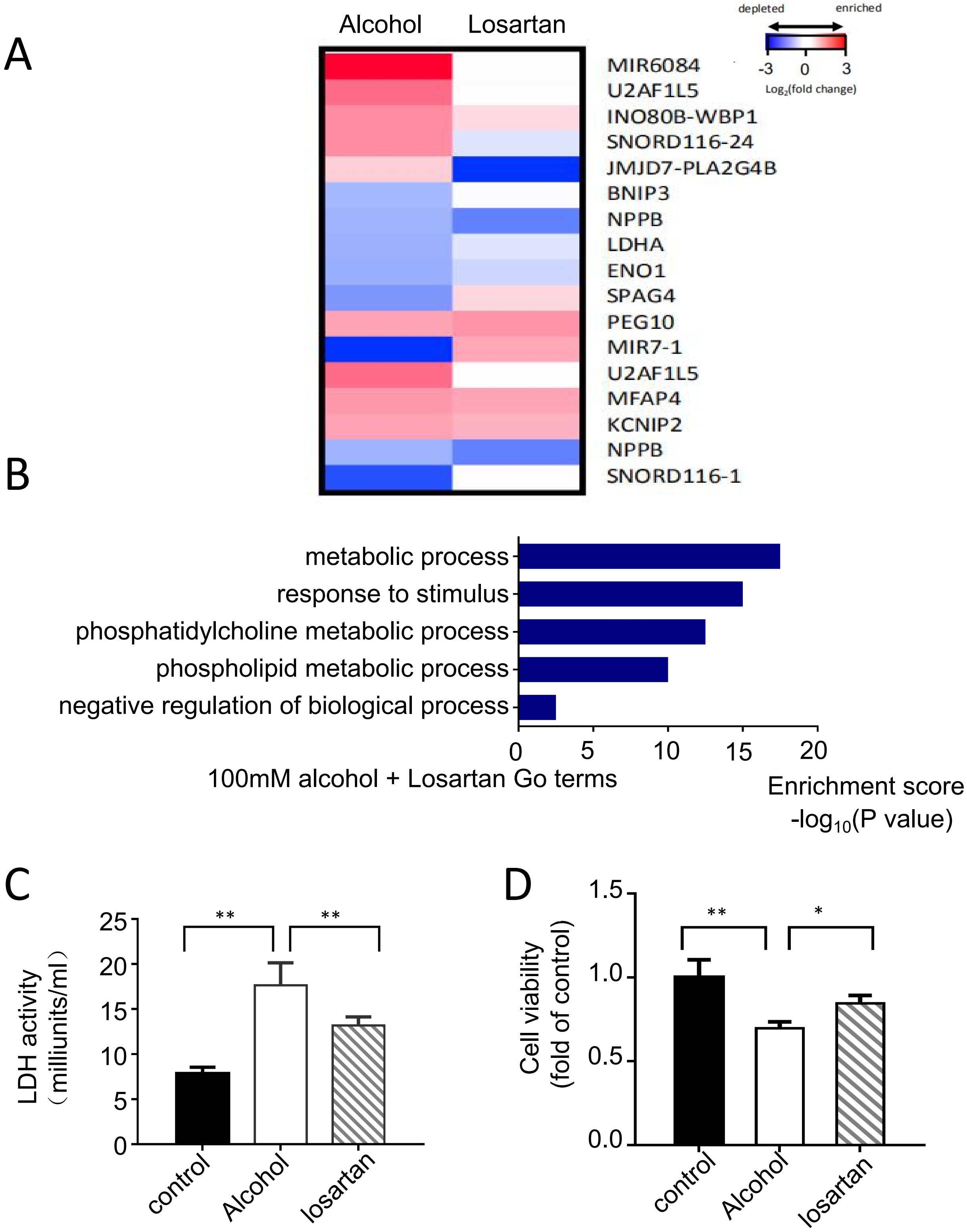
A
Alcohol
Losartan
depleted
enriched
-3
0
3
Log2(fold change)
MIR6084
U2AF1L5
INO80B-WBP1
SNORD116-24
JMJD7-PLA2G4B
BNIP3
NPPB
LDHA
ENO1
SPAG4
PEG10
MIR7-1
U2AF1L5
MFAP4
KCNIP2
NPPB
SNORD116-1
B
metabolic process
response to stimulus
phosphatidylcholine metabolic process
phospholipid metabolic process
negative regulation of biological process
0
5
10
15
20
100mM alcohol + Losartan Go terms
Enrichment score
-log10(P value)
C
LDH activity
（milliunits/ml）
25
20
15
10
5
0
**
**
control
Alcohol
losartan
D
Cell viability
(fold of control)
1.5
1.0
0.5
0.0
**
*
control
Alcohol
losartan

NOX4
(Fig.4D)

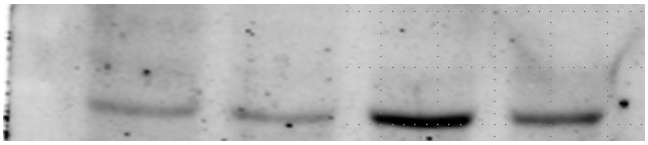

GAPDH
(Fig.4D)

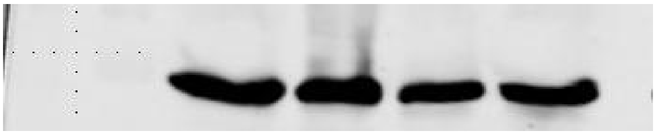

NOX4
(Fig.5D)

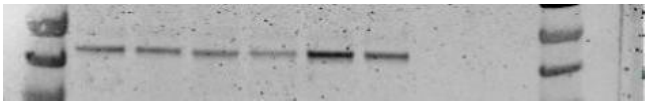

GAPDH
(Fig.5D)

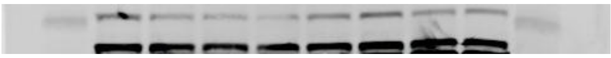

P-P38
(Figure.S2D)

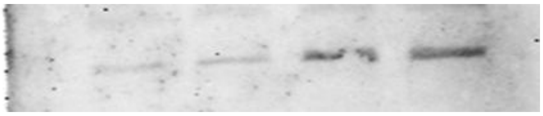

P38
(Figure.S2D）

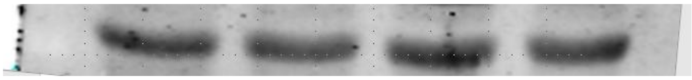

GAPDH
(Figure.S2D）

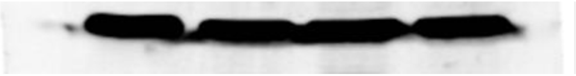

A

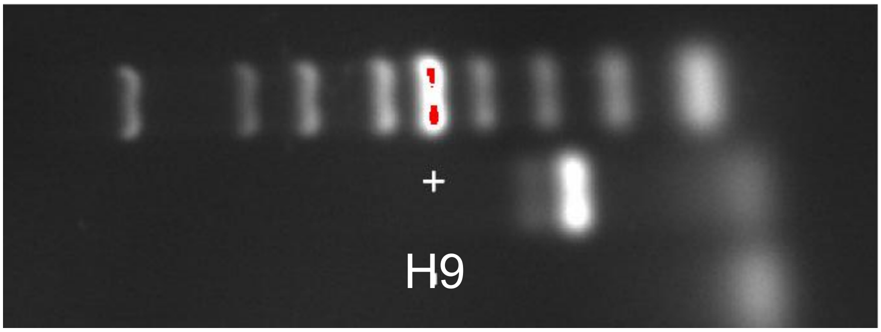
+
H9

H9 STR

B

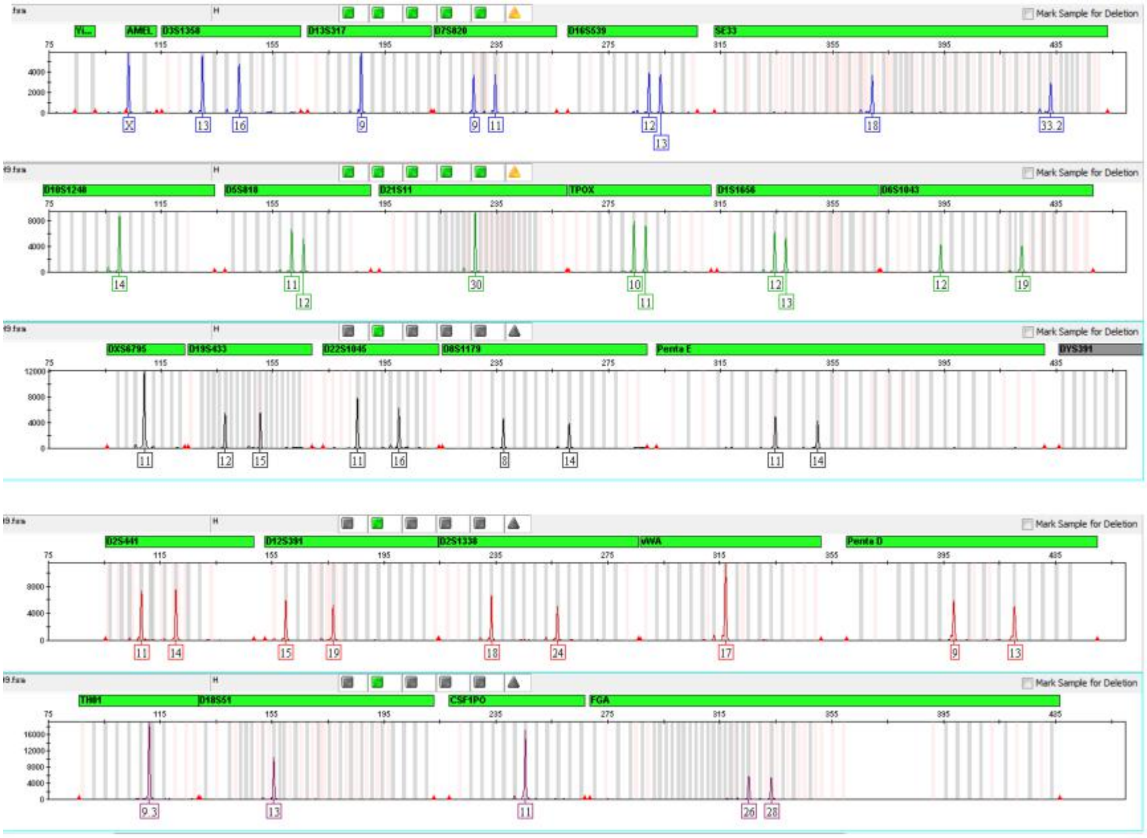
AMEL
D3S1358
D13S317
D7S820
D16S539
SE33
D10S1248
D5S818
D21S11
TPOX
D1S1656
D6S1043
DXS6795
D19S433
D22S1045
D8S1179
Penta E
DYS391
D2S441
D12S391
D2S1338
vWA
Penta D
TH01
D18S51
CSF1PO
FGA
